# Correction: A highly active Z-scheme SnS/Zn_2_SnO_4_ photocatalyst fabricated for methylene blue degradation

**DOI:** 10.1039/d2ra90117j

**Published:** 2022-11-18

**Authors:** Yingjing Wang, Fen Xu, Lixian Sun, Yaying Li, Lumin Liao, Yanxun Guan, Jianhao Lao, Yukai Yang, Tianhao Zhou, Yu Wang, Bin Li, Kexiang Zhang, Yongjin Zou

**Affiliations:** Guangxi Key Laboratory of Information Materials, Guangxi Collaborative Innovation Center for Structure and Properties for New Energy and Materials, School of Material Science and Engineering, Guilin University of Electronic Technology Guilin 541004 P. R. China xufen@guet.edu.cn sunlx@guet.edu.cn

## Abstract

Correction for ‘A highly active Z-scheme SnS/Zn_2_SnO_4_ photocatalyst fabricated for methylene blue degradation’ by Yingjing Wang *et al.*, *RSC Adv.*, 2022, **12**, 31985–31995, https://doi.org/10.1039/D2RA05519H.

The authors regret that an incorrect version of Fig. 1 was included in the original article. The correct version of Fig. 1 is presented below.

**Fig. 1:**
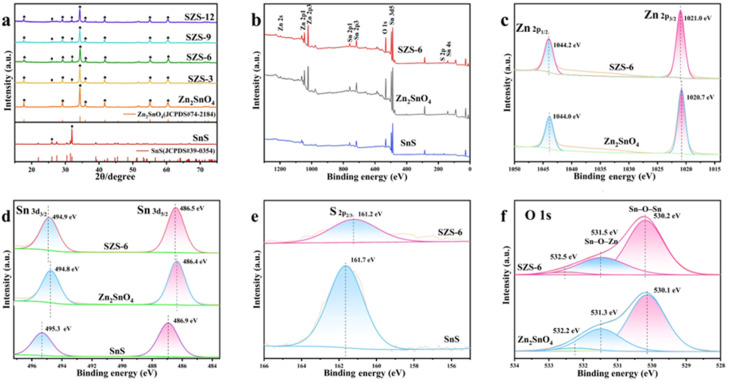
(a) XRD spectra, (b) XPS survey, (c) Zn 2p, (d) Sn 3d, (e) S2p and (f) O 1s spectra of samples.

The authors regret that there was an error in the text in lines 5–10 in the right column on page 31987 of the original article. The text originally read, “The binding energy at 530.1 eV and 531.3 eV belongs to the oxygen atom coordinated with a metal atom (Sn–O–Sn) and (Sn–O–Zn), respectively.^37^ The peak at 531.3 eV is attributed to oxygen in absorbed water, and the binding energy at 532.2 eV is attributed to the oxygen atoms on defect atoms.^37^” This text should read, “The binding energy at 530.1 eV and 531.3 eV corresponds to the oxygen atom coordinated with a metal atom (Sn–O–Sn) and (Sn–O–Zn),^37^ and the binding energy at 532.2 eV is attributed to the hydrated species O–H.^36^”

The Royal Society of Chemistry apologises for these errors and any consequent inconvenience to authors and readers.

## Supplementary Material

